# Longitudinal assessment of liver stiffness and risk factors for advanced fibrosis in adolescents with metabolic dysfunction-associated steatotic liver disease: a retrospective observational study

**DOI:** 10.3389/fmed.2025.1657933

**Published:** 2025-10-01

**Authors:** Wenshu Cao, Congcong Feng, Jizhong Ye, Jianfeng Zhou, Lin Wang, Yanling Lian

**Affiliations:** Tianlin Community Health Center of Xuhui District, Shanghai, China

**Keywords:** metabolic dysfunction-associated steatotic liver disease, vibration-controlledtransient elastography, liver stiffness measurements, longitudinal assessment, adolescents

## Abstract

**Background:**

The global incidence of metabolic dysfunction-associated steatotic liver disease (MASLD) in adolescents is steadily increasing. This research aims to characterize adolescents diagnosed with MASLD clinically and evaluate their long-term outcomes in community and tertiary medical centers located in Shanghai. Furthermore, the investigation assesses the diagnostic efficacy of vibration-controlled transient elastography (VCTE) among adolescents with MASLD.

**Methods:**

We retrospectively analyzed data from adolescent patients (10–18 years) diagnosed with MASLD referred to Shanghai hospitals during the period 2019–2023. Diagnostic criteria included sustained alanine transaminase elevations exceeding twice the upper normal threshold or radiological confirmation of hepatic steatosis, following exclusion of alternative etiologies. VCTE-derived liver stiffness measurements (LSMs) were classified as normal (≤7.0 kPa, F0-F1), significant fibrosis (7.1–9.0 kPa, F2), and advanced fibrosis (≥9.1 kPa, F3-F4), to distinguish fibrosis severity.

**Results:**

A total of 140 adolescents were enrolled (67.9% male), with an average age of 13.8 years. Dyslipidemia was common (48.6%; *n* = 68), followed by impaired glucose tolerance or diabetes (27.1%; *n* = 38) and hypertension (21.4%; *n* = 30). Following a mean follow-up duration of 2.1 years, remission of MASLD occurred in only 8.6% of patients (*n* = 12). Among the 50 patients evaluated by VCTE, 22 (44%) exhibited normal LSM values, whereas significant fibrosis and advanced fibrosis were suspected in 11 (22%) and 17 (34%) individuals, respectively. Independent risk factors significantly associated with advanced fibrosis included age ≥16 years (OR, 7.18), presence of IGT/DM (OR, 10.16), and elevated aspartate aminotransferase levels exceeding 70 U/L (OR, 17.33).

**Conclusion:**

There is a rapid increase in adolescent MASLD incidence in Shanghai. According to LSM assessments, adolescents diagnosed with MASLD may have heightened risks of advanced hepatic fibrosis as they approach late adolescence.

## 1 Introduction

Metabolic dysfunction-associated steatotic liver disease (MASLD), currently affecting approximately 25% of people globally, is now recognized as a prevalent chronic liver disorder and a leading cause of liver transplantation among adults (1, 2). Increasing prevalence of childhood obesity has contributed to rising adolescent MASLD incidence in both Western and Asian populations (3, 4). Recent studies indicate MASLD as the leading chronic liver condition among adolescents in North America and Europe (5, 6). Longitudinal studies have shown that adolescent MASLD can progress through fibrosis and cirrhosis to end-stage liver disease (7).

Histopathological analysis remains the gold standard for diagnosing and staging MASLD, crucially differentiating between simple steatosis and severe netabolic Dysfunction-Associated Steatohepatitis (MASH), thus predicting progression risk toward fibrosis and cirrhosis (8, 9). Alanine aminotransferase (ALT), despite its widespread use as an indirect marker, has limited precision in assessing MASLD subtype and severity (10). Growing emphasis has been placed on noninvasive diagnostic methods, such as clinical scoring models, biochemical assessments, and vibration-controlled transient elastography (VCTE), commonly applied in adults (11). Although validation studies are available (12, 13), these approaches still require additional confirmation in adolescent populations and, according to North American Society for Pediatric Gastroenterology, Hepatology and Nutrition (NASPGHAN) guidelines, have not yet received universal endorsement for routine pediatric application.

The primary aim of this study is to delineate clinical presentations and long-term clinical outcomes in adolescents diagnosed with MASLD who were managed at community and tertiary hospitals in Shanghai from 2019 to 2023. A secondary objective involves evaluating the utility of VCTE in assessing disease severity and the extent of hepatic fibrosis among adolescents with MASLD.

## 2 Methods

### 2.1 Trial design, participants and recruitment

This was a retrospective observational study with longitudinal follow-up of patients over a mean of 2.1 years, involving biannual evaluations and annual VCTE. This analysis included 140 adolescents diagnosed with MASLD, referred from Tianlin Community Health Center to Shanghai Sixth People’s Hospital during the years 2019 to 2023. Ethical approval for this investigation was obtained from the Ethics Committee of Tianlin Community Health Center (Approval number: 2024-KY-017(K)). Eligible patients were aged 10 to 18 years. Hepatic steatosis confirmed by imaging, either ultrasound or computed tomography. The ALT levels persistently exceeding twice the upper limit of normal for more than 3 months. Presence of at least one cardiometabolic risk factor, including overweight or obesity (Overweight and obesity were defined using age- and sex-specific BMI Z-scores: overweight >1 to ≤ 2 SD, obesity >2 SD), dyslipidemia, impaired glucose tolerance or diabetes, or hypertension (2). Exclusion of all other primary liver diseases, determined by clinical history and appropriate diagnostic investigations. This definition is consistent with Chinese guidelines and pediatric evidence indicating that persistent ALT levels exceeding twice the upper limit of normal reliably predict histological steatosis of at least 5%, thereby supporting early detection in community-based practice (2). Exclusion criteria encompassed failure to meet diagnostic standards outlined by the updated Chinese MASLD Prevention and Treatment Guidelines (2018), non-adherence to medical guidance during hospitalization (including irregular medication or premature discharge), and incomplete clinical records such as missing historical data or ≥3 missed follow-up visits.

### 2.2 Data collection

Baseline laboratory values were obtained at initial referral, coinciding with diagnostic ultrasound; VCTE assessments began annually thereafter. Patient dietary patterns and lifestyle behaviors were modified according to established clinical guidelines. Each patient underwent a biannual outpatient evaluation that included anthropometric data collection, physical examination, and hepatic biochemical testing. Follow-up blood tests were standardized for all patients during outpatient evaluations. BMI Z-scores were additionally documented for every participant. The BMI Z-score represents a standardized measure reflecting the deviation of an individual’s BMI from average values matched by age and sex. Because the BMI Z-score adjusts for age- and sex-specific variations, it is crucial for accurately assessing growth and nutritional status in adolescent populations (14). Remission of MASLD was defined, according to pediatric guidelines, as normalization of ALT levels to below the upper limit of normal and resolution of hepatic steatosis on follow-up ultrasonography.

All VCTE was performed using the FibroScan® 502 Touch device (Echosens, Paris, France). This model was consistently used for all patients throughout the study period to ensure uniformity. Annual VCTE assessments were conducted for all patients starting in 2019, measuring liver stiffness (LSM) and controlled attenuation parameters (CAP). Patients fasted for at least 3 h prior to examination and were positioned supine with the right arm elevated. Probe selection (M probe for thoracic perimeter ≤75 cm or XL probe for >75 cm) was based on patient anthropometrics to optimize signal quality. Measurements were obtained in the right intercostal space during brief apnea, targeting a minimum of 10 valid acquisitions per session, with a success rate >60% and interquartile range/median <30% as quality criteria. Invalid measurements were excluded, and the median LSM value was used for analysis. These protocols align with manufacturer guidelines and established clinical standards for VCTE in pediatric populations, as supported by studies demonstrating high reliability in adolescents with MASLD (15). Based on the standards of the Tianlin Community Health Service Center, liver stiffness was categorized as normal (F0-F1, ≤7.0 kPa), significant fibrosis (F2, 7.1–9.0 kPa), and advanced fibrosis (F3-F4, ≥9.1 kPa). These institutional thresholds were derived from studies focusing on pediatric populations. These studies demonstrated that, due to differences in hepatic compliance and body composition between children and adults, pediatric LSM cut-offs at equivalent fibrosis stages are generally 1–3 kPa lower than those in adults (15, 16). Validation against histopathology has confirmed the diagnostic accuracy of VCTE in adolescents with MASLD. CAP thresholds of 248, 268, and 280 dB/m corresponded, respectively, to steatosis severity of ≥5–10% (S1), ≥33% (S2), and ≥66% (S3). These thresholds have been validated against histology in pediatric MASLD (15).

Comorbidities were identified at baseline, or during follow-up if newly developed, according to established criteria: dyslipidemia was defined by fasting lipid profiles (e.g., triglycerides >150 mg/dL); hypertension by repeated blood pressure readings above the 95th percentile for age, sex, and height; and impaired glucose tolerance/diabetes by oral glucose tolerance testing or HbA1c, consistent with American Academy of Pediatrics guidelines (2). Medications were recorded at baseline and each follow-up visit. Vitamin E was prescribed based on clinical suspicion of MASH (persistent enzyme elevation and steatosis) in non-diabetic patients, aligning with trial evidence despite limited pediatric support (17).

### 2.3 Data analysis

Patient demographic characteristics, clinical histories, laboratory findings, imaging data, histological results, and VCTE measurements were extracted from medical records upon referral and at the latest follow-up visit. Incidence rates were calculated as new MASLD diagnoses per 1,000 adolescent referrals in each half-year period, derived from referral logs. Statistical analyses were conducted utilizing IBM SPSS Statistics (Version 22; IBM Corp., Armonk, NY, United States). Continuous variables were summarized as means (standard deviation, SD) or medians with corresponding ranges, while categorical variables were presented as counts and percentages. Comparisons between continuous variables were performed with either the t-test or Mann–Whitney U test; categorical variables were analyzed via χ^2^ or Fisher’s exact tests as appropriate. Stratified analyses compared groups using χ^2^ tests for categorical variables and multivariate logistic regression adjusting for confounders (e.g., age, BMI). A *p*-value less than 0.05 was considered statistically significant.

## 3 Results

### 3.1 Study participants

**Figure 1:**
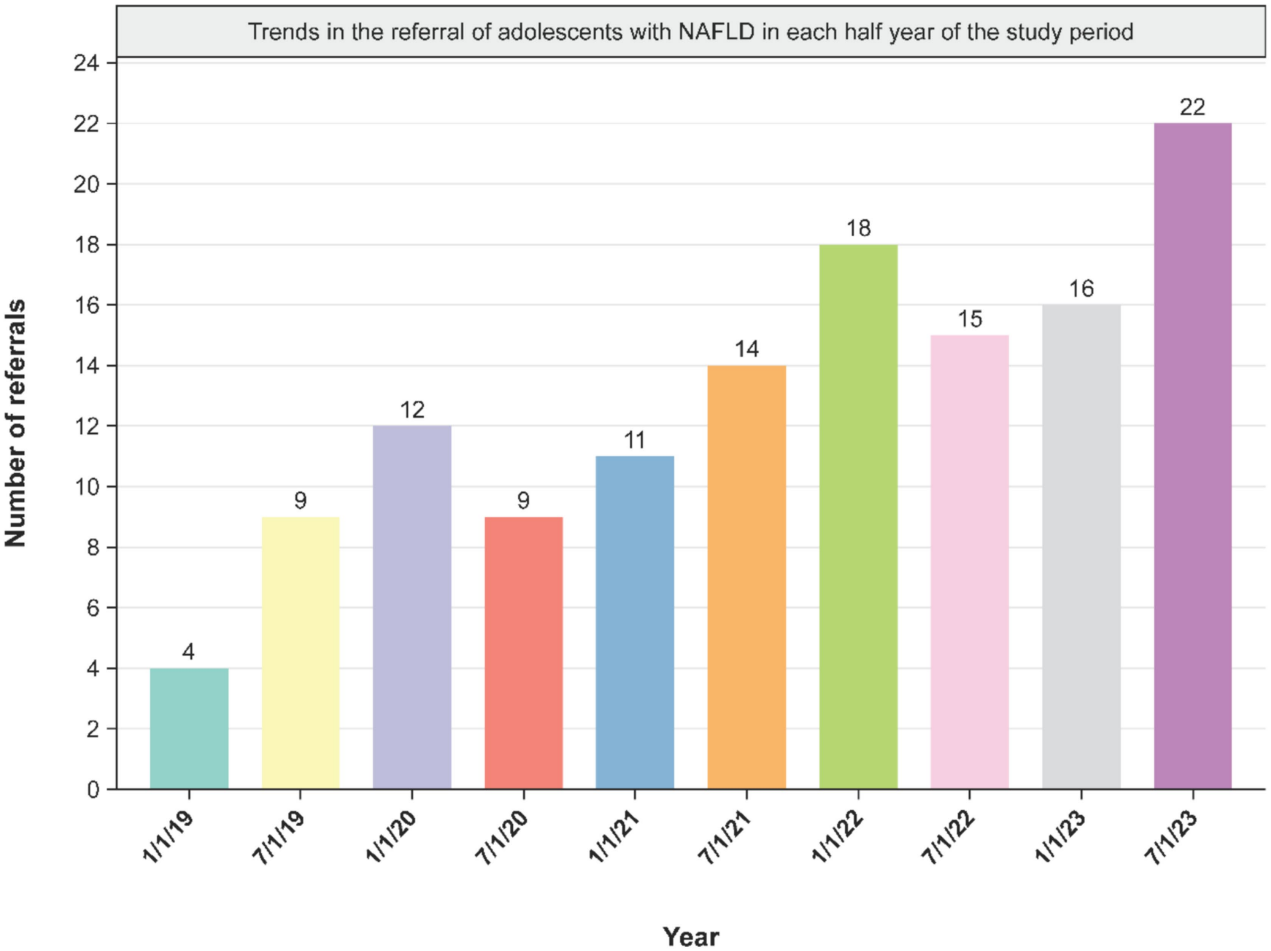
Trends in the number of referrals and incidence of metabolic dysfunction-associated steatotic liver disease in adolescents in each half-year of the study period.

A total of 140 adolescents diagnosed with MASLD were managed during the study period. Among these patients, males constituted 67.9% (*n* = 95), with an average age of 13.8 ± 3.10 years and mean BMI of 28.3 ± 5.81 kg/m^2^. In this cohort, 15 patients (10.7%) were overweight, while obesity was noted in 102 individuals (72.9%). At initial referral, 10 patients (7.1%) were neither overweight nor obese; however, by the time of follow-up, 80% of these had developed increased BMI values (Table 1). The number of newly identified MASLD cases showed a progressive increase throughout the observation interval. Based on monitoring conducted every 6 months, incidence rates per 1,000 referrals increased from 8.5 in 2019–2020, to 14.5 during 2021–2022, and reached 19.5 in 2023 (Figure 1).

**Table 1:** Baseline patient characteristics and comorbidities.

Characteristics	*N* = 140
Mean age (years)	13.8 ± 3.10
Male (%)	95 (67.9)
Mean body mass index (kg/m^2^)	28.3 ± 5.81
Mean body mass index Z-scores	1.8 ± 0.9
Comorbid conditions	
Dyslipidemia (%)	68 (48.6)
Obstructive sleep apnea (%)	34 (24.3)
Hypertension (%)	30 (21.4)
Subclinical abnormal glucose tolerance (%)	21 (15)
Diabetes (%)	17 (12.1)
Hyperuricemia (%)	13 (9.3)
Gastroesophageal reflux or dyspepsia (%)	11 (7.9)
Asthma (%)	14 (10)

### 3.2 Complications

The primary comorbidities included dyslipidemia (*n* = 68, 48.6%), obstructive sleep apnea (*n* = 34, 24.3%), and hypertension (*n* = 30, 21.4%). Additionally, impaired glucose tolerance was documented in 21 patients (15%), and diabetes was identified in 17 patients (12.1%). In total, 72 patients (51.4%) exhibited at least one comorbidity, including dyslipidemia, elevated blood glucose, or hypertension (Table 1).

### 3.3 Initial laboratory test data

Baseline laboratory characteristics for all patients are presented in Table 2. During follow-up, elevated ALT levels (≥80 U/L) occurred in 85 patients (60.7%). Hepatic steatosis was confirmed via ultrasonography in the entire patient cohort (*n* = 140). Liver biopsies were conducted in only four cases, all confirming MASH: two patients had mild fibrosis (F0-F1), one exhibited moderate fibrosis (F2), and another presented with cirrhosis (F4).

**Table 2:** The initial laboratory testing data.

Parameter	Mean (95% CI)
Total protein (g/L)	75.6 (74.3–76.9)
Albumin (g/L)	40.3 (38.9–41.7)
Total bilirubin (μmol/L)	12.1 (11.0–13.3)
Direct bilirubin (μmol/L)	3.9 (3.5–4.3)
Alkaline phosphatase (U/L)	220.3 (209.2–233.4)
ALT(U/L)	120.6 (105.7–135.5)
AST(U/L)	76.6 (65.3–87.9)
Glutamyl transpeptidase (U/L)	60.9 (51.2–70.5)
Total cholesterol (mmol/L)	4.51 (3.24–5.78)
HDL (mmol/L)	1.15 (0.99–1.31)
Triglycerides (mmol/L)	1.47 (1.38–1.56)
LDL (mmol/L)	3.01 (2.94–3.08)
Fasting blood glucose level (mmol/L)	5.1 (4.5–5.7)
Glucose at 120 min (mmol/L)	7.7 (6.9–8.5)
Fasting insulin levels (mIU/mL)	31.5 (26.8–36.1)
Glycated hemoglobin (μmol/L)	479.4 (425.4–533.4)

ALT, Alanine aminotransferase; AST, aspartate aminotransferase; HDL, high-density lipoprotein; LDL, low-density lipoprotein.

### 3.4 Treatment and follow-up

Supplementary medications prescribed by clinicians comprised Vitamin E in 21 cases (15%), ursodeoxycholic acid in 4 cases (2.9%), and probiotics in 11 cases (7.9%). Additionally, metformin was given to 23 individuals (16.4%) for diabetes management, orlistat was used in 10 individuals (7.1%) for obesity treatment, and lipid-lowering medications were provided to 6 individuals (4.3%). During an average follow-up duration of 2.1 ± 1.3 years, 68 patients (49%) demonstrated a notable reduction in BMI Z-scores, with a mean decrease of 0.21 (95% CI: −0.28 to −0.15, *p* < 0.0001) from initial referral to final assessment. Only 12 patients (8.6%) achieved complete resolution of MASLD. Liver enzyme abnormalities normalized in 32 patients (22.9%); however, hepatic impairment persisted in 91 patients (65%). At study completion, 65 individuals (46.4%) continued under active surveillance, 40 individuals (28.6%) ceased follow-up, 20 individuals (14.3%) transferred to adult medical services, and 15 individuals (10.7%) were discharged from care.

### 3.5 Stratified analysis

Fifty patients underwent VCTE, with median liver stiffness measurements (LSM) recorded at 7.4 kPa (range: 3.6–77 kPa). Among them, normal liver stiffness (≤7 kPa) appeared in 22 individuals (44%), significant fibrosis (7.1–9.0 kPa) in 11 (22%), and advanced liver disease (>9.0 kPa) in 17 (34%). After categorization based on age at final follow-up, the proportions of patients younger than 16 years showing normal stiffness, significant fibrosis, and advanced disease were 52.2, 34.8, and 13.0%, respectively. In older adolescents aged 16 or above, these proportions changed notably to 37.0% normal stiffness, 1.1% significant fibrosis, and 51.9% advanced disease (*p* = 0.0095; χ^2^ = 9.312). Additional analysis comparing patients with LSM greater than 9.0 kPa to those with values of 9.0 kPa or lower revealed strong associations between advanced fibrosis and older age, obesity, impaired glucose metabolism, dyslipidemia, as well as elevated levels of ALT, AST, and gamma-glutamyl transferase (Table 3). Multivariate logistic regression analysis adjusting for confounding variables revealed significantly increased risk for advanced fibrosis with age ≥16 years (OR, 7.18; 95% CI, 1.72–29.98; *p* = 0.004), presence of impaired glucose tolerance or diabetes mellitus (OR, 10.16; 95% CI, 2.57–40.16; *p* < 0.001), and AST levels exceeding 70 U/L (OR, 17.33; 95% CI, 3.87–77.72; *p* < 0.001; Table 3).

**Table 3:** Univariate comparison of clinical and biochemical characteristics between patients with LSM ≤ 9 kPa and those with LSM > 9.0 kPa, indicating an increased likelihood of advanced fibrosis.

Index	LSM≤9.0 kPa (*n* = 33)	LSM>9.0 kPa (*n* = 17)	*T or χ* ^2^	*p* value
Age (years)	14.12 ± 3.01	16.51 ± 2.12	2.608	0.002
Age ≥16 years (%)	13 (39.4)	14 (82.4)	8.336	0.004
Male (%)	19 (57.6)	10 (58.8)	0.007	0.933
Gastroesophageal reflux or dyspepsia (%)	8 (24.2)	13 (76.4)	12.564	<0.001
Dyslipidemia (%)	10 (30.3)	9 (52.9)	2.441	0.118
Hypertension (%)	7 (21.2)	8 (47.1)	3.569	0.059
Obstructive sleep apnea (%)	6 (18.2)	6 (35.3)	1.801	0.294
Abnormal total bilirubin (%)	4 (12.1)	3 (17.6)	0.285	0.594
Mean ALT (U/L)	108.86 ± 67.92	178.45 ± 70.23	3.356	0.005
ALT>140 U/L (%)	7 (21.2)	13 (76.4)	14.275	<0.001
Mean AST (U/L)	58.76 ± 32.34	118.19 ± 45.76	4.776	0.001
AST > 70 U/L (%)	7 (21.2)	14 (82.4)	17.218	<0.001
Mean GGT(U/L)	54.47 ± 28.98	88.32 ± 34.15	3.490	0.015
GGT > 45 U/L (%)	14 (42.4)	13 (76.4)	5.236	0.022

ALT, Alanine aminotransferase; AST, aspartate aminotransferase; GGT, Glutamyl transpeptidase.

## 4 Discussion

The prevalence of adolescent MASLD in China has notably increased from 2003 onward. Shanghai, a highly developed municipality in China, has a resident population exceeding 20 million people. Increased awareness and expanded MASLD screening efforts in recent years reflect significant advancements in clinical practice. However, due to the asymptomatic and silent nature of MASLD, many community members continue to be undiagnosed. Our results revealed that multiple metabolic abnormalities, including impaired glucose tolerance, dyslipidemia, and elevated ALT, AST, and GGT, were present in adolescents starting at a young age. Analysis of liver stiffness measurements by VCTE identified independent predictors of advanced fibrosis, specifically age ≥16 years, impaired glucose tolerance or diabetes, and AST levels greater than 70 U/L. These findings are essential for optimizing early detection methods for adolescent MASLD, enhancing diagnostic accuracy, and initiating community-level interventions to prevent hepatic progression.

Epidemiological trends observed among Shanghai adolescents with MASLD are consistent with global data. MASLD continues to be the predominant chronic liver disorder among adolescents, with an estimated prevalence ranging from 5 to 10% in this demographic group. In East Asia, MASLD prevalence among adolescents in South Korea increased from 7.8% (2001–2005) to 11.2% (2015–2017), coinciding with rising obesity rates (18). Zhang et al. (19) indicated a global annual increase in MASLD prevalence of around 1.35% among adolescents and young adults from 1990 to 2017, with the steepest increase occurring in North America. These global findings align closely with our observations of increasing adolescent MASLD prevalence in Shanghai. Rapid urbanization, unhealthy dietary habits, and decreased physical activity among adolescents significantly contribute to these trends, making MASLD an increasingly pressing public health issue. Therefore, enhancing nutritional education, promoting physical exercise, and conducting active community-based MASLD screening programs are crucial preventive strategies.

Metabolic syndrome continues to significantly influence the development of MASLD in younger populations. Previous research indicates that overweight or obese adolescents diagnosed with metabolic syndrome, characterized by dyslipidemia, poor glucose regulation, and hypertension, exhibit substantially greater risks of biopsy-proven MASLD compared with unaffected peers (20–22). Our findings similarly confirm strong associations among obesity, impaired glucose metabolism, dyslipidemia, and severe hepatic fibrosis in MASLD. It is noteworthy that our cohort exhibited a higher proportion of diabetes (12.1%) compared to the previously reported range of 5–10% in other studies (18, 23). This discrepancy might originate from the recruitment across two distinct centers, potentially leading to selection bias. Although insulin-resistant impaired glucose regulation may critically connect MASLD to metabolic syndrome, it remains unclear whether dyslipidemia induced by hepatic lipid accumulation is a cause or consequence of the metabolic disturbance. Additionally, increased blood pressure in adolescents merits greater consideration, given that pediatric metabolic syndrome significantly raises future risks of diabetes, arterial conditions, and cardiovascular diseases (24, 25).

Adolescent MASLD often presents with a phenotype more severe than that typically observed in adults. Research indicates severely obese adolescents have an 83% prevalence of hepatic fibrosis, significantly exceeding the 29% prevalence in adults, and typically exhibit more advanced stages of MASH on histopathology (26). Retrospective cohort studies have found that fibrosis is already present in 34.7–80% of adolescents undergoing liver biopsy, with subsequent biopsies commonly indicating further stage advancement (27, 28). In the control arm of one clinical trial involving 122 participants undergoing repeat biopsies, MASH was observed in 31%, and fibrosis of stage 2 or greater was noted in 29% (29, 30). These findings indicate that, over an average follow-up duration of 1.6 years, histological progression in MASH or fibrosis stage occurred in 36% of cases. Furthermore, only three participants (2.4%) achieved resolution of MASLD, consistent with the 8.6% resolution rate observed in our study, despite BMI reduction in almost half the cohort. Our longitudinal observations further suggest adolescent MASLD progresses rapidly, is typically severe, and frequently takes an irreversible course.

The VCTE is now a key noninvasive modality for assessing disease severity in adolescents with MASLD. In adults, the World Federation for Ultrasound in Medicine and Biology (WFUMB) proposed the “Rule of 5 s” for transient elastography, in which liver stiffness values <5 kPa indicate normal findings, 5–10 kPa exclude advanced fibrosis, 10–15 kPa suggest advanced fibrosis, and >15 kPa strongly support advanced fibrosis (31). Existing studies also demonstrate that VCTE predicts histological fibrosis in adolescents with MASLD with high accuracy. However, these thresholds were developed for adults, and reference ranges for pediatric and adolescent populations are generally lower. The thresholds applied in our study align closely with recent pediatric evidence; for instance, a US adolescent cohort identified ≥7.4 kPa as the diagnostic cut-off for fibrosis, supporting the validity of our chosen values in adolescent populations (32). A separate study demonstrated a strong correlation between CAP measurements from VCTE and hepatic steatosis severity; notably, CAP ≥259 dB/m could detect grades 1–3 steatosis with 94% sensitivity and 91% specificity (33). In our study, longitudinal monitoring of LSM and CAP via VCTE among adolescents further emphasizes its practical clinical utility in managing MASLD. These findings possess significant clinical implications, supporting VCTE as an effective noninvasive diagnostic method for adolescent MASLD.

This study has several limitations, including retrospective data collection, potential selection bias, and a relatively high rate of participant attrition. Only four participants underwent liver biopsy, limiting the ability to directly correlate VCTE results with histological findings. This limitation mainly resulted from adolescents’ reluctance toward routinely undergoing invasive procedures. Despite the absence of precise incidence data, this study represents the largest reported cohort of adolescents with MASLD in Shanghai. These findings provide important clinical and epidemiological insights into adolescent MASLD patterns within Shanghai and eastern China.

In conclusion, adolescent MASLD prevalence in Shanghai, China, has notably increased over the past 20 years, accompanied by parallel rises in obesity-related metabolic and cardiovascular complications. In younger populations, MASLD represents a chronic liver condition capable of progression, sometimes resulting in severe fibrosis by late adolescence.

## Data Availability

The original contributions presented in the study are included in the article/supplementary material, further inquiries can be directed to the corresponding authors.
